# 2,5-Diamino­thio­phene-3,4-dicarbonitrile

**DOI:** 10.1107/S1600536812034678

**Published:** 2012-08-11

**Authors:** Christopher J. Ziegler, Victor N. Nemykin

**Affiliations:** a Department of Chemistry, University of Akron, Akron, OH 44325-3601, USA; b Department of Chemistry & Biochemistry, University of Minnesota Duluth, Duluth, MN 55812, USA

## Abstract

In the title compound, C_6_H_4_N_4_S, the planar mol­ecule lies across a crystallographic mirror plane. In the crystal, the mol­ecules form centrosymmetric dimers through cyclic amino N—H⋯N hydrogen-bonding associations with cyano N-atom acceptors [graph set *R*_2_^2^(12)] and these dimers are extended through amine–cyano N—H⋯N associations into a three-dimensional network.

## Related literature

For the synthesis of this and related compounds *via* the reaction of tetra­cyano­ethyl­ene with hydrogen sulfide, see: Cairns *et al.* (1957 ▶); Middleton *et al.* (1958 ▶); Middleton (1959 ▶). For the use of this compound as a reagent, see: Nemykin *et al.* (2012 ▶). For graph-set analysis, see: Etter *et al.* (1990 ▶). For details of the weighting scheme, see: Prince (1982 ▶); Watkin (1994 ▶).
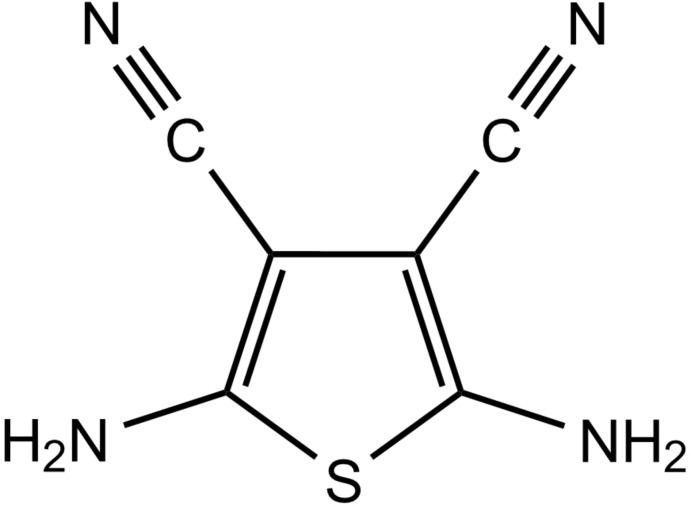


## Experimental

### 

#### Crystal data

C_6_H_4_N_4_S
*M* *_r_* = 164.19
Orthorhombic, 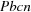

*a* = 3.9231 (2) Å
*b* = 13.8213 (12) Å
*c* = 12.6465 (11) Å
*V* = 685.72 (9) Å^3^
*Z* = 4
Mo *K*α radiation
μ = 0.40 mm^−1^
*T* = 123 K
0.41 × 0.24 × 0.16 mm

#### Data collection

Rigaku RAPID II diffractometer
Absorption correction: ψ scan (North *et al.*, 1968 ▶) *T*_min_ = 0.69, *T*_max_ = 0.94
2260 measured reflections
783 independent reflections
492 reflections with *I* > 2σ(*I*)
*R*_int_ = 0.054

#### Refinement

*R*[*F* ^2^ > 2σ(*F* ^2^)] = 0.053
*wR*(*F* ^2^) = 0.116
*S* = 0.99
769 reflections
51 parameters
H-atom parameters constrained
Δρ_max_ = 0.65 e Å^−3^
Δρ_min_ = −0.63 e Å^−3^

### 

Data collection: *CrystalClear* (Rigaku, 2009 ▶); cell refinement: *HKL-2000* (Otwinowski & Minor, 1997 ▶); data reduction: *CrystalClear*; program(s) used to solve structure: *SHELXS86* (Sheldrick, 2008 ▶); program(s) used to refine structure: *CRYSTALS* (Betteridge *et al.*, 2003 ▶); molecular graphics: *CAMERON* (Watkin *et al.*, 1996 ▶); software used to prepare material for publication: *CRYSTALS*.

## Supplementary Material

Crystal structure: contains datablock(s) global, I. DOI: 10.1107/S1600536812034678/zs2223sup1.cif

Structure factors: contains datablock(s) I. DOI: 10.1107/S1600536812034678/zs2223Isup2.hkl

Supplementary material file. DOI: 10.1107/S1600536812034678/zs2223Isup3.cml

Additional supplementary materials:  crystallographic information; 3D view; checkCIF report

## Figures and Tables

**Table 1:** Hydrogen-bond geometry (Å, °)

*D*—H⋯*A*	*D*—H	H⋯*A*	*D*⋯*A*	*D*—H⋯*A*
N1—H2⋯N2^i^	0.87	2.29	3.106 (5)	156
N1—H1⋯N2^ii^	0.88	2.38	3.196 (5)	153

Symmetry codes: (i) 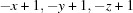; (ii) 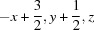.

**Fig. 1.**
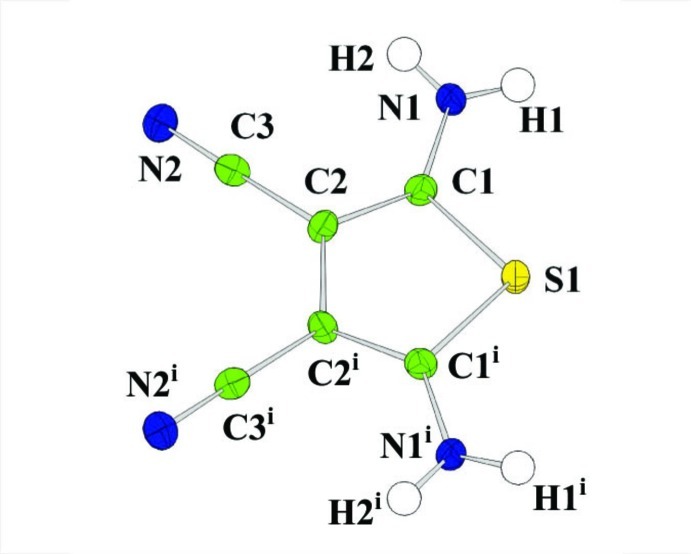
The title compound with displacement ellipsoids drawn at the 50% probability level. H atoms are shown as spheres of arbitary radius. For symmetry code (i): -x+2, y, -z+3/2.

## supplementary crystallographic information

### Comment

The synthesis of the title compound 2,5-diamino-3,4-dicyanothiophene, C_6_H_4_N_4_S, and similar compounds has previously been reported (Cairns *et al.*, 1957; Middleton, 1959) and chemical transformations of this compound and its usage in macrocyclic chemistry have also been described (Middleton *et al.*, 1958; Nemykin *et al.*, 20120. In the structure of the title compound the planar molecule lies across a crystallograpic mirror plane (Fig. 1). The C—S bond length is 1.750 (4) Å and the C—C bond distances are unequal [1.358 (5) Å for C1—C2 and 1.458 (7) Å for C2—C2^i^ [for symmetry code (i): -*x*+2, *y*, -*z*+3/2]. The C—N_amine_ bond distance [1.358 (5) Å] shows some double-bond character and the C2—C3 bond length [1.422 (5) Å] is shorter than expected for a single bond. The cyanide C3—N2 bond length is 1.149 (5) Å.

In the crystal, the molecules form centrosymmetric cyclic dimers through amino N—H···N hydrogen-bonding associations with cyano N-atom acceptors (Table 1) [graph set *R*^2^_2_(12) (Etter *et al.*, 1990)] and these dimers are extended into a three-dimensional structure through N—H···N amine···cyano group associations. The thiophene molecules form antiparallel stacks down *a*, with a thiophene-thiophene ring centroid separation of 3.923 (2) Å.

### Experimental

The title compound was prepared using an earlier published procedure via the reaction of tetracyanoethylene and hydrogen sulfide (Cairns *et al.*, 1957) and characterized by ^1^H and ^13^C NMR spectroscopy. The single crystal used for the X-ray analysis was obtained by slow cooling of a saturated solution in DMSO.

### Refinement

The H atoms were all located in a difference map. The H atoms were initially refined with soft restraints on the bond lengths and angles to regularize their geometry (N—H in the range 0.86–0.89 Å) and *U*_iso_(H) (in the range 1.2–1.5 times *U*_eq_ of the parent atom), after which the positions were refined with riding constraints. In the absence of significant anomalous scattering, Friedel pairs were merged.

### Crystal data

**Table:**

C_6_H_4_N_4_S	*D*_x_ = 1.590 Mg m^−^^3^
*M**_r_* = 164.19	Melting point: 513 K
Orthorhombic, *P**b**c**n*	Mo *K*α radiation, λ = 0.71073 Å
Hall symbol: -P 2n 2ab	Cell parameters from 783 reflections
*a* = 3.9231 (2) Å	θ = 2–27°
*b* = 13.8213 (12) Å	µ = 0.40 mm^−^^1^
*c* = 12.6465 (11) Å	*T* = 123 K
*V* = 685.72 (9) Å^3^	Plate, brown
*Z* = 4	0.41 × 0.24 × 0.16 mm
*F*(000) = 336	

### Data collection

**Table:**

Rigaku RAPID II diffractometer	783 independent reflections
Radiation source: Mo Ka	492 reflections with *I* > 2σ(*I*)
Graphite monochromator	*R*_int_ = 0.054
ω scans	θ_max_ = 27.5°, θ_min_ = 3.4°
Absorption correction: ψ scan (North *et al.*, 1968)	*h* = −5→5
*T*_min_ = 0.69, *T*_max_ = 0.94	*k* = −13→17
2260 measured reflections	*l* = −13→16

### Refinement

**Table:**

Refinement on *F*^2^	Primary atom site location: structure-invariant direct methods
Least-squares matrix: full	Hydrogen site location: inferred from neighbouring sites
*R*[*F*^2^ > 2σ(*F*^2^)] = 0.053	H-atom parameters constrained
*wR*(*F*^2^) = 0.116	Method, part 1, Chebychev polynomial, (Watkin, 1994; *P*rince, 1982) [*w*eight] = 1.0/[A_0_*T_0_(x) + A_1_*T_1_(x) ··· + A_n-1_]*T_n-1_(x)] where A_i_ are the Chebychev coefficients listed belo*w* and x = *F* /*F*max Method = Robust Weighting (*P*rince, 1982) W = [*w*eight] * [1-(delta*F*/6*sigma*F*)^2^]^2^ A_i_ are: 57.9 74.5 21.5
*S* = 0.99	(Δ/σ)_max_ = 0.0000992
769 reflections	Δρ_max_ = 0.65 e Å^−^^3^
51 parameters	Δρ_min_ = −0.63 e Å^−^^3^
0 restraints	

### Special details

**Table:**

Experimental. The crystal was placed in the cold stream of an Rigaku XStream 2000 open-flow nitrogen cryostat with a nominal stability of 0.1 K.

### Fractional atomic coordinates and isotropic or equivalent isotropic displacement parameters (Å^2^)

**Table:**

	*x*	*y*	*z*	*U*_iso_*/*U*_eq_	
S1	1.0000	0.75078 (10)	0.7500	0.0189	
C1	0.8517 (10)	0.6628 (3)	0.6618 (3)	0.0184	
N1	0.7035 (9)	0.6908 (2)	0.5697 (2)	0.0205	
H2	0.6326	0.6473	0.5246	0.0500*	
H1	0.6790	0.7530	0.5557	0.0500*	
C2	0.9146 (10)	0.5716 (3)	0.6988 (3)	0.0188	
C3	0.8190 (10)	0.4873 (3)	0.6416 (3)	0.0211	
N2	0.7390 (11)	0.4203 (2)	0.5938 (3)	0.0286	

### Atomic displacement parameters (Å^2^)

**Table:**

	*U*^11^	*U*^22^	*U*^33^	*U*^12^	*U*^13^	*U*^23^
S1	0.0243 (7)	0.0137 (5)	0.0188 (6)	0.0000	−0.0041 (6)	0.0000
C1	0.0202 (19)	0.0184 (17)	0.0166 (16)	−0.0027 (16)	0.0025 (15)	−0.0009 (13)
N1	0.0285 (19)	0.0152 (14)	0.0177 (14)	0.0003 (14)	−0.0060 (14)	0.0012 (11)
C2	0.023 (2)	0.0153 (16)	0.0181 (17)	−0.0018 (15)	0.0016 (16)	−0.0027 (14)
C3	0.024 (2)	0.0213 (19)	0.0176 (17)	0.0006 (17)	−0.0003 (16)	0.0021 (15)
N2	0.040 (2)	0.0205 (16)	0.0249 (17)	−0.0053 (17)	−0.0034 (18)	−0.0021 (13)

### Geometric parameters (Å, º)

**Table:**

S1—C1^i^	1.750 (4)	N1—H1	0.884
S1—C1	1.750 (4)	C2—C2^i^	1.458 (7)
C1—N1	1.358 (5)	C2—C3	1.422 (5)
C1—C2	1.367 (5)	C3—N2	1.149 (5)
N1—H2	0.874		
			
C1^i^—S1—C1	91.9 (3)	H2—N1—H1	120.2
S1—C1—N1	119.4 (3)	C2^i^—C2—C1	112.8 (2)
S1—C1—C2	111.3 (3)	C2^i^—C2—C3	125.0 (2)
N1—C1—C2	129.3 (3)	C1—C2—C3	122.3 (3)
C1—N1—H2	120.0	C2—C3—N2	178.6 (4)
C1—N1—H1	119.8		

Symmetry code: (i) −*x*+2, *y*, −*z*+3/2.

### Hydrogen-bond geometry (Å, º)

**Table:**

*D*—H···*A*	*D*—H	H···*A*	*D*···*A*	*D*—H···*A*
N1—H2···N2^ii^	0.87	2.29	3.106 (5)	156
N1—H1···N2^iii^	0.88	2.38	3.196 (5)	153

Symmetry codes: (ii) −*x*+1, −*y*+1, −*z*+1; (iii) −*x*+3/2, *y*+1/2, *z*.
